# Tumor necrosis factor prevents *Candida albicans* biofilm formation

**DOI:** 10.1038/s41598-017-01400-4

**Published:** 2017-04-26

**Authors:** Francisco Airton Castro Rocha, Anelise Maria Costa Vasconcelos Alves, Marcos Fábio Gadelha Rocha, Rossana de Aguiar Cordeiro, Raimunda Sâmia Nogueira Brilhante, Ana Carolina Matias Dinelly Pinto, Rodolfo de Melo Nunes, Virgínia Cláudia Carneiro Girão, José Julio Costa Sidrim

**Affiliations:** 1 Department of Internal Medicine, Faculty of Medicine, Federal University of Ceará, Fortaleza, Brazil; 2 Specialized Medical Mycology Center, Federal University of Ceará, Fortaleza, Ceará Brazil; 3 Department of Morphology, Faculty of Medicine, Federal University of Ceará, Fortaleza, Brazil

## Abstract

*Candida* species are commensals but some develop biofilms in prosthetic materials and host surfaces that may represent up to 30% of deaths related to infections, particularly in immunosuppressed patients. Tumor necrosis factor (TNF) exhibits a plethora of functions in host defense mechanisms whereas excessive release of TNF in inflammation promotes tissue damage. Cytokines released in an inflammatory milieu may influence the development of microorganisms either by promoting their growth or displaying antimicrobial activity. In protozoa, TNF may affect growth by coupling through a lectin-like domain, distinct from TNF receptors. TNF was also shown to interact with bacteria via a mechanism that does not involve classical TNF receptors. Using an *in vitro C. albicans* biofilm model, we show that TNF dose-dependently prevents biofilm development that is blocked by incubating TNF with *N*,*N*’-diacetylchitobiose, a major carbohydrate component of *C. albicans* cell wall. This finding represents a relevant and hitherto unknown mechanism that adds to the understanding of why TNF blockade is associated with opportunistic *C. albicans* infections.

## Introduction

Biofilms are the major state that microorganisms utilize in their struggle to thrive since antimicrobials act against their planktonic, free-floating, state. Development of aggregates formed by the microorganism and an extracellular coat of secreted components and even host parts leads to biofilm formation^1^. In biofilm development, features related to the microorganism and to the extracellular polymeric substances that compose its environment are relevant^2^.

Patients subjected to immunosuppression as well as those exposed to implanted devices and indwelling catheters are particularly affected by life-threatening systemic infections. Organization in biofilms after adherence to those devices offers to microorganisms an alternative to evade host defense. *Candida albicans* account for a major part of those opportunistic infections with a mortality rate that can reach 40% of affected individuals, with obvious health and economic impacts^3^.

There is extensive knowledge on the role of cytokines in human defense against microbes. Usually, cytokines are synthesized and released by host cells after being triggered by diverse stimuli. Analogous to other cytokines, TNF acts on mammalian cells via coupling to specific membrane or soluble receptors, leading to cell activation. Excessive TNF release during inflammation is associated to pain development, cell infiltration, hypotension, hepatotoxicity, and structural damage to tissues^4–6^. Thus, targeting TNF has become an alternative to treat autoimmune diseases, including rheumatoid arthritis and inflammatory bowel disease. Considering that TNF has a major protective role against microorganisms, anti-TNF treatment would render patients prone to develop life-threatening infections^4^.

To the best of our knowledge, TNF was never shown to directly provoke changes in fungal development without the participation of mammalian cells. However, there is evidence that TNF induces trypanosome lysis through a TNF receptor independent mechanism^7^. We investigated whether TNF could alter *C. albicans in vitro* growth. Our data, showing a previously unrecognized TNF interference with *C. albicans* biofilm formation, unravels a protective role of TNF against systemic, life-threatening opportunistic infections. In addition to the importance in the pathogenesis of *Candida* infections, this finding does also offer an alternative to prevent yeast biofilm formation.

## Results

### TNF alters *C. albicans* biofilm metabolism

**Figure 1:**
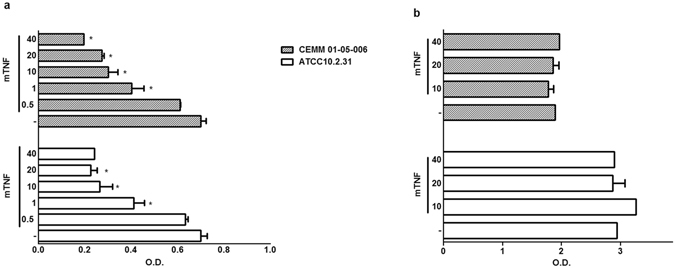
Effect of TNF on biofilm formation by *C. albicans*. Murine tumor necrosis factor (mTNF ng/mL) or medium (−) were added to growing (**a**) or mature (**b**) biofilms of two strong biofilm producers *C. albicans* strains. Biofilm metabolic activity was assessed using the XTT reduction assay. Data represent the mean ± SEM (Absorbance_492nm_) of two independent experiments conducted in triplicate; *P < *0.001* as compared to medium.

Adding increasing concentrations of TNF to the growing biofilm of two strong biofilm producer strains of *C. albicans* (ATCC 10.2.31; CEMM 01-005-006) significantly and dose-dependently inhibited the metabolic activity of the yeast biofilm, measured by the 2,3-bis(2-methoxy-4-nitro-5-sulfophenyl)- 2H-tetrazolium-5-carboxanilide (XTT) assay (Fig. 1a). The assay evaluates yeast metabolic ability to reduce XTT leading to a water-soluble formazan-colored product. The results, originally designed to evaluate *Candida* biofilms, provide a semiquantitative estimation of the metabolic activity of the biofilm^8^. On the other hand, TNF solutions that affected growing *C. albicans* biofilm did not alter mature biofilm metabolic activity (Fig. 1b), suggesting that the cytokine has no effect in an established biofilm. Further, adding TNF to the same *C. albicans* strains in a free-living, planktonic condition, did not influence yeast growth so that readings obtained adding TNF were similar to those after incubation solely with medium (RPMI). As a control of antifungal activity, amphotericin B significantly impaired *C. albicans* planktonic growth (data not shown).

### TNF alteration of yeast morphology

**Figure 2:**
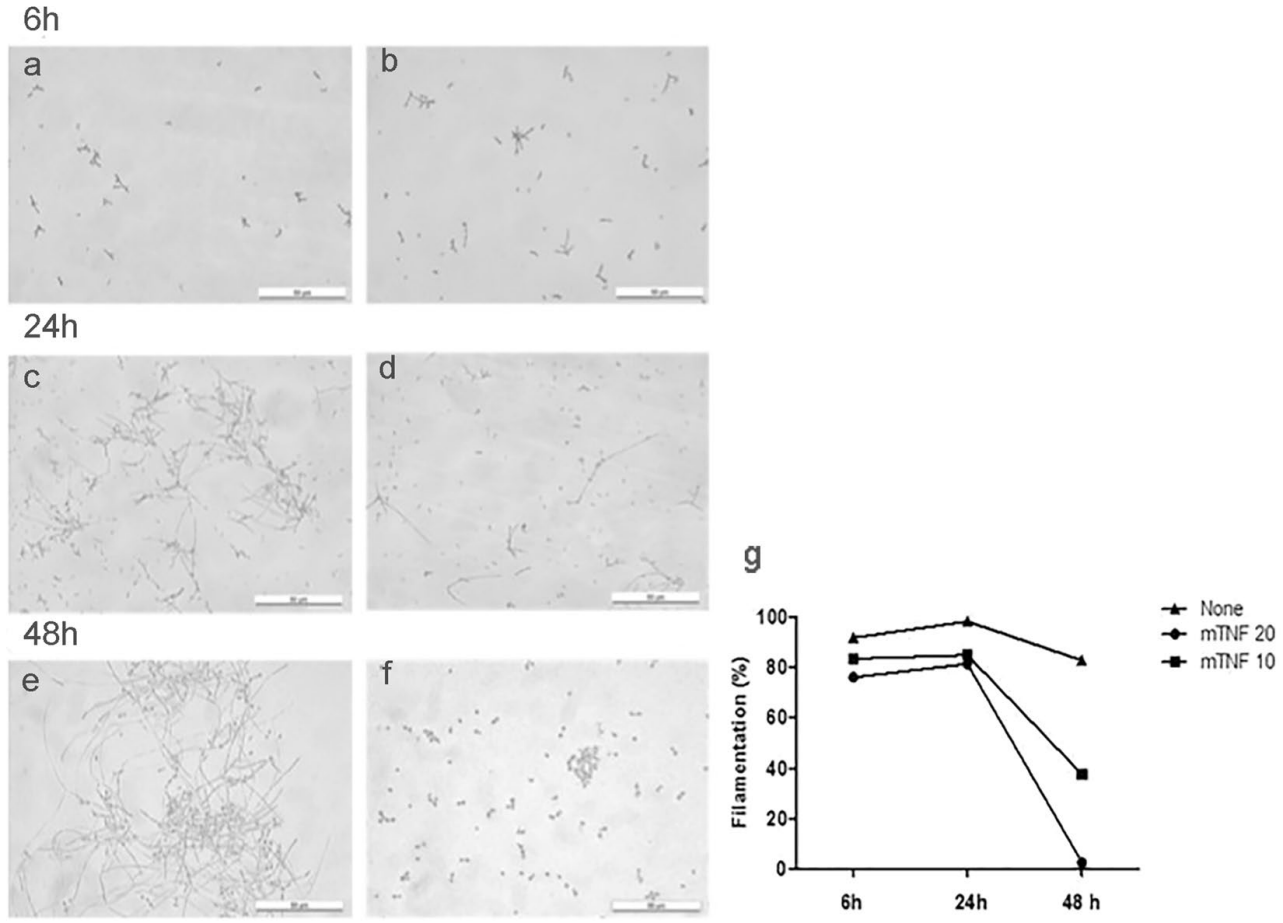
Effect of TNF on the morphology of preformed *C. albicans biofilm*. Illustrations of lactophenol cotton blue dye stained *C. albicans* ATCC 10.2.31 growing biofilms treated with TNF (20 ng/mL) (**b**,**d**,**f**) or untreated (**a**,**c**,**e**) after 6, 24 or 48 h incubation (Original x200); (**g**) *C. albicans* ATCC 10.2.31 growing biofilms were treated with mTNF (ng/mL) or untreated (none) for 6, 24 or 48 h. Filamentation index represents the percentage of hyphae/yeast measured in 10 high power fields, under inverted optical microscopy.

**Figure 3:**
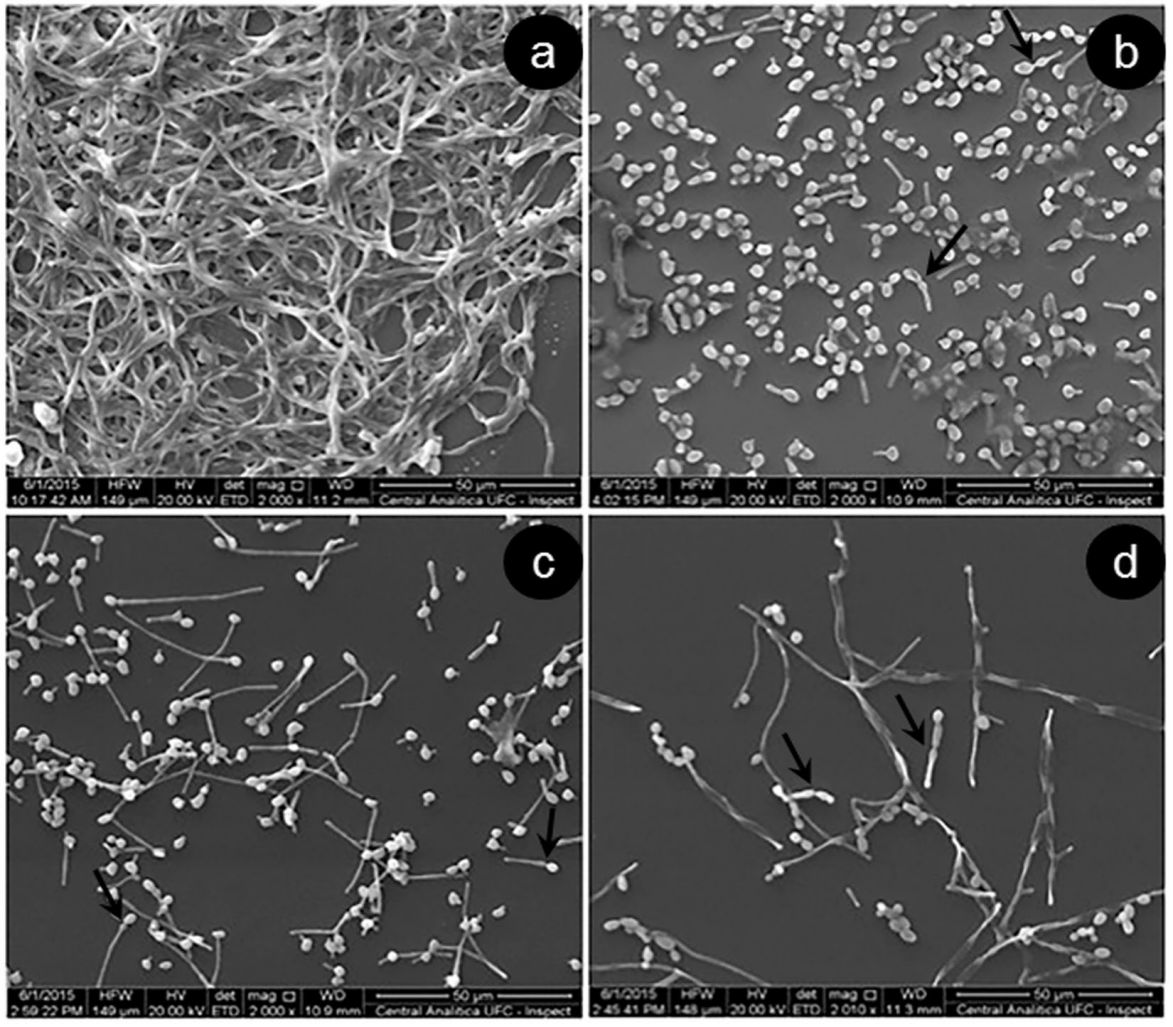
Scanning electron microscopy of *C. albicans* ATCC 10.2.31 growing biofilms after 48 h incubation left untreated (**a**) or treated with 0.1, 10, or 20 ng/mL mTNF (**b**,**c**,**d**), respectively. Arrows indicate pseudohyphae (Original x2000).

Morphological evaluation and blastoconidia filamentation (yeast to hyphal development) of *C. albicans* TNF-treated biofilms is shown in Fig. 2a–g. TNF addition to *C. albicans* growing biofilm led to a decreased filamentation after 24 h of incubation. The appearance, as well as the filamentation index, after 6 h of incubation (Fig. 2a,b) is similar in TNF-treated and untreated biofilms, whereas 24 h and 48 h after incubation there was a significant reduction in the number of hyphae, thus suggesting a TNF interference with yeast filamentation and biofilm maturation (Fig. 2d,f,g). On the other hand, untreated *C. albicans* biofilm showed a clear formation of branched hyphae (Fig. 2c,e). Increasing TNF concentrations apparently impaired the formation of true hyphae, so that only blastoconidia and pseudohyphae were observed under scanning electron microscopy, while untreated biofilms demonstrated a typical, dense structure, with yeast, pseudohyphae, and hyphae (Fig. 3a,d).

### Effects of anti-TNF compounds

**Figure 4:**
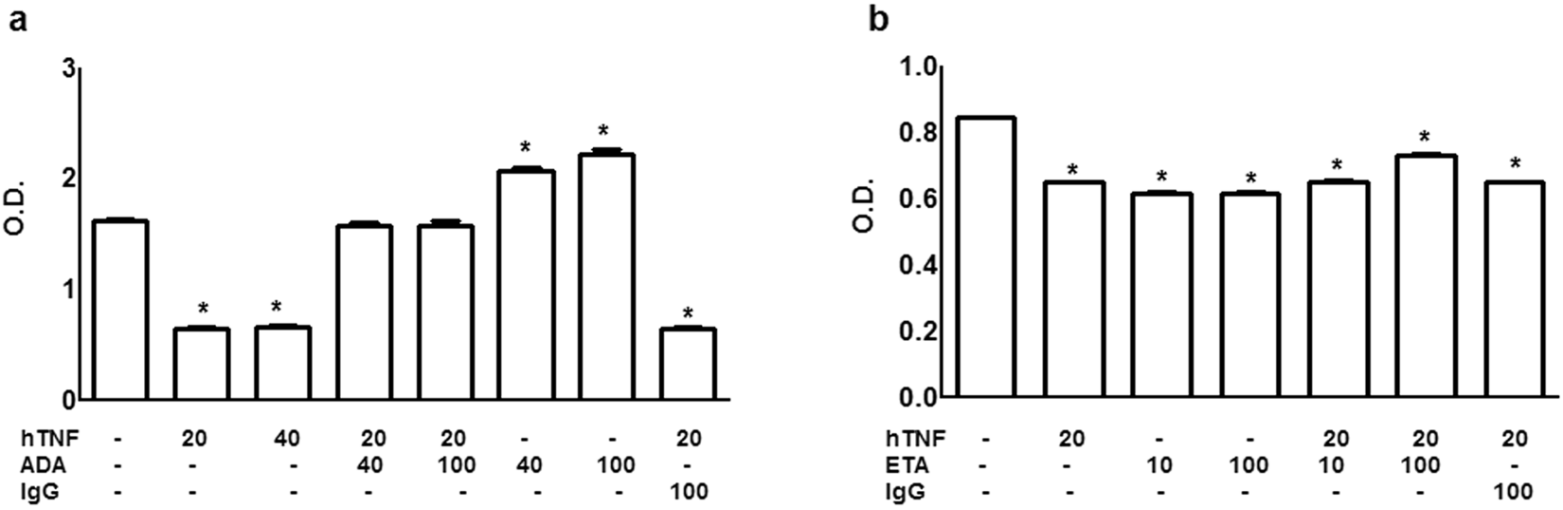
Anti-TNF compounds effect on *C. albicans* biofilm. Human TNF (hTNF ng/mL), the anti-TNF monoclonal antibody Adalimumab (ADA ng/mL), the IgG coupled soluble TNF-receptor Etanercept (ETA ng/mL), human immunoglobulin G (IgG) or (−) Medium were added isolated or combined to *C. albicans* ATCC 10.2.31 growing biofilm. The biological activity was assessed using the XTT reduction assay. Data represent the mean ± SEM Absorbance_492nm_ of two independent experiments conducted in triplicate; *P < *0.001* as compared to medium.

Although *Candida* species have not been shown to display classic TNF receptors, we wondered whether TNF inhibitors would alter that TNF effect on *Candida* biofilms. Preincubation of TNF with the humanized monoclonal anti-TNF antibody adalimumab inhibited TNF effect on growing biofilms, whereas adding adalimumab without TNF actually increased biofilm metabolic activity, when compared to TNF-untreated biofilm (Fig. 4a). On the other hand, pre-incubation with the IgG coupled soluble TNF receptor etanercept did not alter TNF effect whereas adding etanercept without TNF significantly decreased biofilm metabolic activity, similar to incubation with TNF alone. Incubation with irrelevant immunoglobulin did not alter TNF effect (Fig. 4a,b).

XTT results display variability between assays. However, we should call attention to the fact that adding murine or human TNF to growing *C. albicans* biofilms consistently and reproducibly decreased biofilm metabolic activity. We may speculate that conformational changes or steric hindrance account for adalimumab blockade of that TNF activity. As expected, incubation with etanercept, presumably blocking TNF access specifically to its classic receptor, did not interfere with *Candida* biofilm thus arguing that the cytokine effect depended on binding via its lectin-like domain. Similar data were shown in fluid reabsorption using *in situ* and *in vivo* flooded rat lungs where TNF activation of lung liquid clearance via its lectin-like domain was inhibited by the anti-TNF antibody infliximab but not by a soluble TNF receptor^9^. However, the inhibition and improvement in biofilm formation provided by etanercept and adalimumab, respectively, are hard to explain. In clinical practice, it has been shown that anti-TNF monoclonal antibodies and etanercept may differ regarding efficacy to treat acute anterior uveitis in patients with ankylosing spondylitis^10^. More recently, the administration of either a monoclonal anti-TNF antibody or a soluble TNF receptor increased mice susceptibility to hematogeneously disseminated candidiasis whereas only the monoclonal antibody increased susceptibility to oropharyngeal candidiasis^11^. Differences in the glycan pattern on therapeutic proteins, including adalimumab and etanercept, affect their binding to lectins and biological activity^12^, opening the possibility of such a mechanism to explain this apparently controversial result.

### A specific oligosaccharide blocks TNF effect

**Figure 5:**
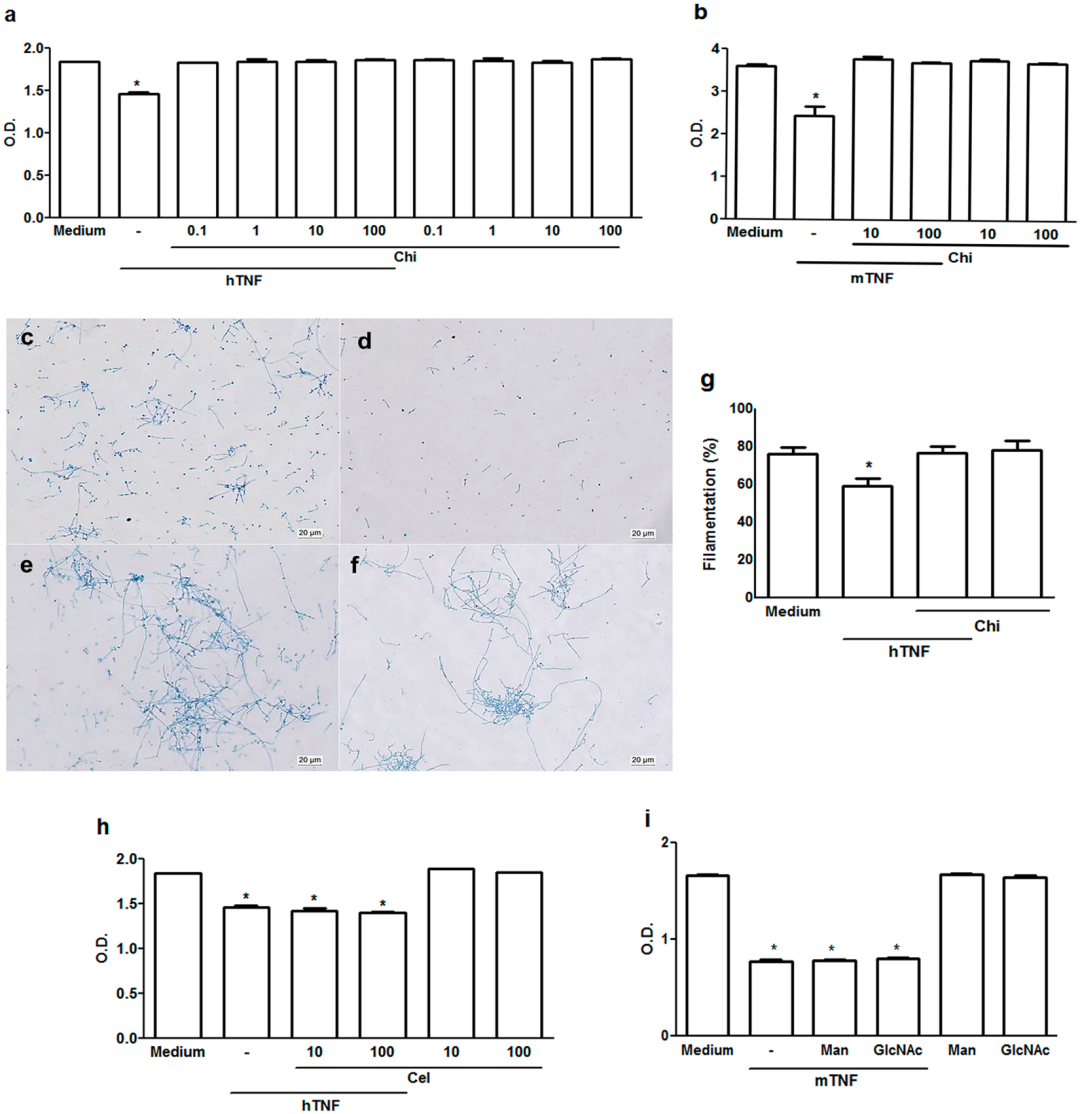
*N,N*-diacetylchitobiose blocks TNF effect on *C. albicans* biofilm. (**a**,**b**) *N,N*-diacetylchitobiose (Chi µg/mL) isolated or combined with hTNF or mTNF (ng/mL) or medium (−) was added to *C. albicans* ATCC 10.2.31 biofilm (metabolic activity − XTT assay); optical microscopy of biofilms grown with medium (**c**), medium + mTNF 20 (**d**), medium + mTNF (20) + Chi (100) (**e**), or medium + Chi (100) (**f**) for 48 h (Original x200); (**g**) filamentation index of biofilms after 48 h growth with medium, medium + hTNF (20), medium + hTNF + Chi (100) or medium + Chi (100); (**i**) metabolic activity after treatment with cellobiose (Cel), mannose (Man) or N-Acetylglucosamine (GlcNAc). Data represent the mean ± SEM Absorbance_492nm_ of two independent experiments conducted in triplicate; *P < *0.001*. Filamentation index represents the percentage of hyphae/blastoconidia in 10 hpf (Original x200).

*N*,*N*′-diacetylchitobiose, a carbohydrate present in *C. albicans* cell wall, was previously shown to block TNF trypanosomicidal activity^7, 13^. Further, *β*-glucan induced TNF release from macrophages was inhibited by *N*,*N*′-diacetylchitobiose showing that the lectin-like domain, which is distinct from TNF residues that bind TNF receptors, is responsible for that cytokine effect^14^. D-glucose, *N*-acetyl-D-glucosamine (GlcNAc), and D-mannose are major carbohydrates in *Candida* cell wall. Polymers of GlcNAc are called chitin and aggregate to glucans (glucose polymers) strengthening the fungus cell wall. A chitobiose is formed by GlcNAc molecules and provides linkage of asparagine to glucan residues in the cell wall. Those carbohydrates are very relevant to yeast metabolism. For instance, the structure of the septum during hyphae formation is formed by chitin. In addition, bioses rather than monosaccharides, as competitors, are considered more relevant to demonstrate a role of carbohydrates in yeast physiology^13^. Preincubation of TNF with *N,N*′-diacetylchitobiose abrogated the cytokine inhibition of biofilm formation, restoring yeast filamentation, a step where carbohydrates participate in *Candida* growth. This was a clear and significant effect shown whether using the XTT assay or biofilm morphological analysis (Fig. 5a–i). It has to be remarked that addition of isolated *N*,*N*′-diacetylchitobiose did not modify biofilm formation. Addition of cellobiose, a disaccharide that does not bind to TNF lectin-like domain^7^, or the monosaccharides mannose or GlcNAc, two other major components of *Candida* cell wall, also did not alter TNF activity (Fig. 5h,i). Similarly, incubation with other nonspecific carbohydrates (D-xylose, arabinose, glucose) did not impair TNF activity on biofilm growth (data not shown).

## Discussion

The present findings, showing an as yet unrecognized mechanism of TNF interference with *C. albicans* biofilm growth are relevant both as the description of a physiological, TNF receptor independent mechanism of this cytokine in host defense against *C. albicans* as well as the opening of a potential strategy to prevent *in vivo Candida* biofilm growth. TNF activity is commonly linked to specific membrane receptors coupling, triggering the activation of downstream cellular pathways thereby inducing the transcription of genes associated with the inflammatory response. Planktonic, free growing *C. albicans* was not affected by TNF leading us to speculate the involvement of a mechanism interfering with the extracellular biofilm matrix. This TNF mechanism is particularly relevant to dissect host barriers against life-threatening infections caused by opportunistic agents. In addition to the reduction of the metabolic activity of growing *C. albicans* biofilms treated with TNF, there was a marked reduction in hyphae filamentation, most likely interfering with cell division. *C. albicans* biofilm formation, which is under a complex gene network regulation, comprises adherence to a surface, followed by proliferation, growth of pseudohyphae and true hyphae, and a later dispersal phase^3, 15^. Extracellular matrix accumulates during biofilm maturation, being essential to its resistance to antimicrobials^2, 15^. Mechanisms involved in TNF inhibition of biofilm growth may include alteration of gene expression or direct interference with extracellular matrix formation. The fact that TNF concentrations used in the present study resemble those used in other *in vitro* TNF studies on mammalian cells indicates that the obtained results likely are clinically relevant.

Trypanosome species, which do also lack classic TNF receptors, are susceptible to lysis via a TNF domain with a lectin-like activity. Substitutions in the amino acid sequence encompassing the Thr104 to Glu109 positions of murine(m) TNF or Thr105 to Glu110 of human(h) TNF altered that lectin-like activity^7^. Additionally, a synthetic peptide bearing the specific TNF sequence that mimics the lectin domain prevented tissue damage in an *in vivo* lung inflammatory model^16^.

TNF is one of the cytokines operating both in innate and adaptive immune mechanisms and our results were similar regardless of using mTNF or hTNF. Other cytokines may also operate similarly. Interleukin(IL)-17 was recently shown to promote *C. albicans* and *Aspergillus fumigatus* planktonic and biofilm growth, activating various genes, rendering those fungi less susceptible to antimicrobials^17^.

Limitations of our study include the use of an *in vitro* biofilm model, as it may not reflect *in vivo* systems. However, patients exposed to treatment with etanercept were reported to have increased susceptibility to oral candidiasis^18^ and it was recently shown that TNF protected mice from disseminated *C. tropicalis* infection^19^. An in depth approach to possible mechanisms to explain TNF effect on yeast metabolism and biofilm structure is also needed to better evaluate the clinical relevance of the present data. Institutionalized patients as well as those needing implanted devices and prosthetic material are seriously affected by systemic, resistant, multigerm infections, commonly due to biofilm formation in implanted materials. Lining of implant materials with bioactive components is a current prophylactic strategy to prevent infections, rendering the development of compounds reproducing TNF domain with that lectin activity to halt *C. albicans* biofilm growth plausible.

## Methods

### Microorganisms

ATCC 10.2.31 and CEMM 01.05.006*C. albicans* strains from the collection of the Specialized Medical Mycology Center (CEMM, Federal University of Ceará, Brazil^19, 20^).

### Reagents

TNF solutions were prepared at the time of use using RPMI 1640 supplemented with L-glutamine. Recombinant rat TNF-α (mTNF) and human Tumor Necrosis Factor-α (hTNF) were purchased from R&D Systems and Sigma-Aldrich, São Paulo, Brazil, respectively. Other reagents were purchased from Sigma-Aldrich do Brasil Ltda., SP, Brazil. Adalimumab and Etanercept were from Abbvie Farmaceutica and Laboratórios Pfizer Ltda., São Paulo, Brazil, respectively.

### *In vitro* susceptibility testing

Susceptibility of *C. albicans* to TNF was determined using the broth microdilution method (Protocol M27-A3; The Clinical and Laboratory Standards Institute - CLSI). Amphotericin B was a drug control.

### *In vitro* biofilm assay

TNF solutions were prepared using buffered RPMI 1640. Biofilms were grown on 96-well microtitre plates. Fungal suspensions containing approximately 0.25 × 10^3^CFU mL^−1^ in RPMI 1640 supplemented or not with TNF solutions, added at the start of the experiment, were incubated for 48 h, at 37 °C to evaluate TNF effect in growing biofilms. In order to investigate TNF effect on mature (preformed) biofilms, fungal suspensions containing approximately 0.25 × 10^3^CFU mL^−1^ in RPMI 1640 were incubated for 48 h, at 37 °C, medium was then aspirated, nonadherent cells removed by thoroughly washing with sterile buffered PBS and wells were then filled with RPMI supplemented or not with TNF solutions and incubated for an additional period of 48 h, at 37 °C. Biofim formation was calculated using XTT (2,3-Bis-(2-Methoxy-4-Nitro-5-Sulfophenyl)- 2H-Tetrazolium-5-Carboxanilide) assay^8^, performed after either 48 h (growing biofilm) or 96 h (preformed biofilm) of incubation. XTT was added and samples were incubated at 36 °C for 5 h using an orbital shaker. After staining, plates were read at 492 nm.

### Biofilm morphological analysis

After 6, 24 or 48 h incubation, plates were stained with lactophenol cotton blue dye and photographed under an inverted light microscope (Fisher Scientific™). The number of filamentous (hyphae) and yeast (blastoconidia) in 10 high power fields (hpf; Original x200) was calculated to obtain a filamentation index: Filamentation = number hyphae/total cell counts (hyphae and blastoconidia) ×100.

### Ultra structural analysis

Biofilms were fixed with Karnovsky solution, dehydrated in graded ethanol, dried in hexamethyldisilazane, coated with gold and observed in an Inspect S50™ scanning electron microscope.

### Statistical analysis

Data are means ± SEM of at least 3 replicates for each treatment, analyzed using one-way ANOVA, followed by Tukey’s test.
